# An open chat with… Laszlo Nagy

**DOI:** 10.1002/2211-5463.13374

**Published:** 2022-02-14

**Authors:** Duncan E. Wright, Laszlo Nagy

**Affiliations:** ^1^ FEBS Open Bio Editorial Office Cambridge UK; ^2^ Departments of Medicine and Biological Chemistry Johns Hopkins University School of Medicine Institute for Fundamental Biomedical Research Johns Hopkins All Children's Hospital St. Petersburg FL USA; ^3^ Department of Biochemistry and Molecular Biology Nuclear Receptor Research Laboratory Faculty of Medicine University of Debrecen Hungary

## Abstract

Laszlo Nagy has been on the Editorial Board of *FEBS Open Bio* since the journal’s inception and is a passionate supporter of FEBS Press and other society journals. Currently, he is also an editor of *FEBS Letters* and *The Journal of Clinical Investigation* (*JCI*). He studied medicine at the University Medical School of Debrecen in Hungary, where he graduated with an M.D. and later Ph.D., and then moved to the United States to conduct postdoctoral research at the University of Texas–Houston and subsequently the Salk Institute in San Diego. Laszlo is a Professor of Medicine and Biological Chemistry at John Hopkins School of Medicine, where he is Co‐Director of the Institute for Fundamental Biomedical Research and Associate Director of the Center for Metabolic Origins of Disease, and Adjunct Professor at the University of Debrecen. Formerly, he was a Professor and Founding Director of the Genomic Control of Metabolism Program at the Sanford Burnham Prebys Medical Discovery Institute. He is also a member of the European Molecular Biology Organisation (EMBO), Academia Europaea, the Hungarian Academy of Sciences and The Henry Kunkel Society, and recipient of several awards, including the Boehringer Ingelheim Research Award, Cheryl Whitlock/Pathology Prize, a Wellcome Trust Senior Research Fellowship in Biomedical Sciences, and three Howard Hughes Medical Institute International Research Scholar Awards. In this fascinating interview, Laszlo Nagy shares the advice that changed his career trajectory, relates his views on scientific publishing, discusses new developments at The Johns Hopkins Center for Metabolic Origins of Disease, and outlines the prospects for future development of research and technology infrastructures in eastern Europe.

## Graphical abstract

Laszlo Nagy has been on the Editorial Board of *FEBS Open Bio* since the journal’s inception. Here, he shares the advice that changed his career trajectory, shares his views on scientific publishing, discusses new developments at The Johns Hopkins Center for Metabolic Origins of Disease, and outlines the prospects for future development of research and technology infrastructures in eastern Europe.

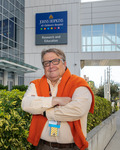

## For those who are non‐specialists, how would you describe the central thread that connects your research?

I am interested in how cells become what they are, that is, how they differentiate and specify, which is a very broad and fundamental biological question. We, as organisms, all start out with one cell, a fertilised oocyte, which gives rise to hundreds of different tissues and cell types. I have been fascinated by the process by which cells, all with the same instruction book, that is our genome, are able to generate all these different cell types which then respond differently to various stimuli. Obviously, over the course of my career I’ve had to narrow this down – I’m not working on embryogenesis or similar very fundamental biological processes, but I’m not unhappy where I am (*laughs*), and that is basically to try to understand how certain signals specify the function of cells via a nuclear hormone receptor and for that I use the macrophage as a model system.

**Figure:**
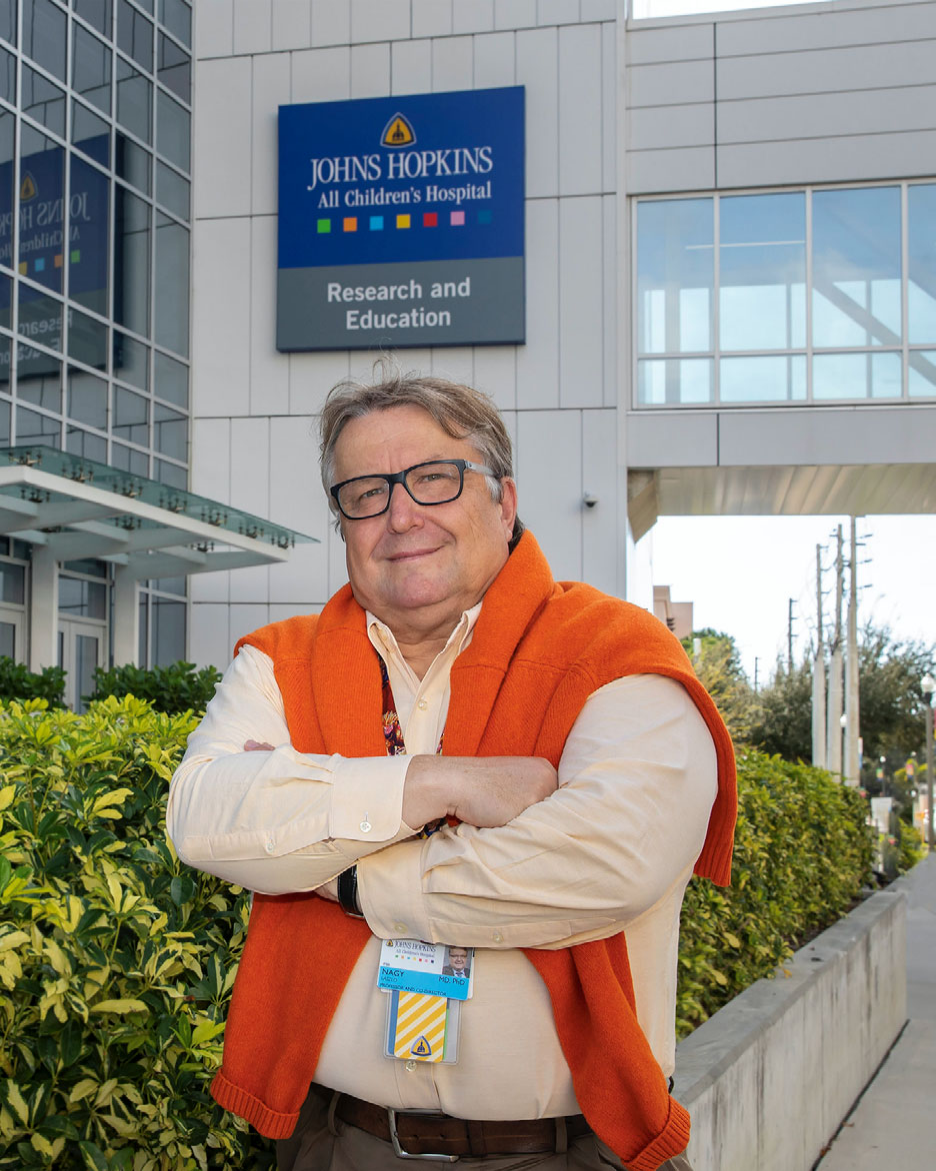
Photo credit: Allyn DiVito/JHACH.

As a little bit of introduction to nuclear hormone receptors – I got engaged with that research when I was a medical student with László Fésüs at the University of Debrecen, when I started to work on retinoids and how retinoids regulate gene expression, and that led me to my first postdoc in the States with Peter Davies at the University of Texas in Houston and then a second with Ronald Evans at the Salk Institute in San Diego. I try to understand how these lipid hormone‐activated transcription factors, which are members of the steroid hormone receptor family, turn on and off genes, and I try to frame my research questions in this context of how a response to a changing extra‐ and intra‐cellular lipid environment instructs a cell to change its gene expression and, as a consequence, its phenotype. Over the years, I developed an appreciation for macrophages, which I call the ‘Swiss Army Knife of the body’, because they are in every tissue and organ, and they have very different functions. To support those different functions, they need different patterns of gene expression, and so I try to find through my research how these changes occur at the level of the genome.

A short answer to the question is: my interest is how cell fates are determined, and my binoculars are focussed on nuclear hormone receptors and macrophages, which are my models.

## Over your career, which discoveries or new technological developments (yours or others) would you say have dramatically impacted on your research?

I can give you two examples:

The first goes back to my years as a medical student at Debrecen: for my thesis, I was carrying out research on the regulation of a gene called *transglutaminase* and its association with a biological process called apoptosis, or programmed cell death. One day, a News and Views article was published in Nature about a pair of papers published in the journal [1], and Professor Fesus put the article on my desk. The title of the News and Views article was ‘We have a morphogen!’ And that morphogen was retinoid acid; the year was 1987, which was when the retinoic acid receptors were identified. And that was particularly momentous, because no one at the time knew how retinoids worked, and certainly, a link between transcriptional regulation, morphogenesis and retinoids had not been made until then. And that was particularly interesting because we were looking at how this particular gene, *transglutaminase*, is regulated by retinoids, and all of a sudden, there was a mechanism which we could explore. So that particular discovery put me on track to be able to work in Houston and later San Diego on the fundamental mechanisms by which lipid‐activated transcription receptors, among them retinoic acid receptors, regulate gene expression and cell fate and responses [2, 3, 4, 5].

The second example, which is perhaps pretty cliché but opened many doors for me, is the sequencing of genomes. I tried to take full advantage of this, as our focus in the field of nuclear hormone receptors was to try to identify the genes regulated by particular transcription factors. There have been different methods, based on candidate approach, to measure some end point and then track back and identify what the receptor does, but it’s remarkably difficult to conclusively show that a particular gene is regulated by a particular transcription factor upon a signal. But now we have sequenced genomes and the technologies that accompany them (i.e. microarrays, RNA‐seq, differential display, ChIP‐seq, other seqs and genome‐wide approaches and so forth) and can use these various assays to identify the chromatin environment and the binding site of these transcription factors. We can now connect those with even newer technologies to find links between promoters and enhancers, which has provided us with the most wonderful tools to accelerate our research. It became an ethos in our laboratory, when we have a biological system, a model which we study, to first have an unbiased exploratory phase, when we catalogue everything that changes upon a signal, or activation of a transcription factor, and then dive into a particular pathway or regulated gene.

So these two events, my fascination with lipids regulating cell fate, in this case vitamin A and retinoic acid receptor, and later PPARg (Peroxisome Proliferator‐Activated Receptor gamma) and the availability of genome‐wide surveys of gene expression and gene regulation, have shaped my career to a large extent.

## What would you consider the greatest unanswered question(s) regarding macrophage function and regulation?

Macrophages are exposed to many different stimuli often simultaneously, which they integrate like a microprocessor [6], so I would be very much interested to find out the governing principles of such signal response and integration events, especially at the chromatin and gene expression level. Using this information I would like to harness the healing power of macrophages, for example in processes such as regenerative inflammation, where macrophages support tissue growth and repair [7].

The other, related question is, how programmable macrophages are? Can we arrive at a point when we can use either small molecules, antibodies, or proteins to program macrophages at will in certain parts of the body in a therapeutically useful way to repair tissues or eliminate unwanted cells such as cancers? There are reports that this may be possible, but nothing is being used in the clinic at present.

## What inspired you to study medicine and ultimately pursue a career in basic science?

It’s not as glamorous as one might think, as it was more a pragmatic choice: I was a sickly child, in retrospect not very serious diseases, but at the time, I found it traumatic that I had to go to the doctor more often than my peers. And I was curious, I wanted to know what was going on with me, so I said: ‘Okay, one way to do it is to become a physician!’ (*laughs*) So it was basically my curiosity about diseases which drove me toward medical school. Further motivation was given by the fact that my mother worked as a pharmacist and my father as a country veterinarian.

Although I’m qualified in medicine I’ve never practised and I’ve not even done a residency. In Hungary, the first two years of medical curricula are very heavily focussed on basic science, and so I got hooked on that when I realised how much biology could be uncovered through research. Ironically, in the third year when we started to study the clinical subjects, including pathology, I realised how little was known about diseases and found it was essentially all descriptive: if it’s green or blue, then it’s this disease, if it’s yellow, it’s that disease. There was no explanation, let alone a molecular explanation, behind the pathology. I decided I wanted to be somebody who studies disease, rather than someone who memorises symptoms and treats patients. I’m a huge advocate of the training of physician‐scientists, MD‐PhD programs, or just having MDs in a basic research laboratory environment, because the insights of MDs into disease processes really help research groups to focus on disease‐oriented research, and I think my medical training combined with my enthusiasm for molecular biology helped my career tremendously.

## You are Associate Director of The Johns Hopkins Center for Metabolic Origins of Disease – do you have any goals for the centre?

Yes, very much so. It’s an unusual, one might call it out of the box, arrangement – John Hopkins University is in Baltimore, while we reside in St. Petersburg, Florida. John Hopkins Medicine acquired All Children’s Hospital and agreed to turn it into a paediatric academic medical centre and as part of it built a beautiful research building where we reside. Our goal for this centre is to populate the five floors of the building; we currently have nine research groups – we started with six – and I and my director colleague, Tim Osborne, have plans to recruit at least another ten research groups in collaboration with the other clinical institutes to reach critical mass and sustainability.

One of the major research foci of the centre is the metabolic origins of diseases, and we are responsible for recruiting the right people, providing them with the right equipment, sharing the core facilities, and ensuring that they integrate well with both the clinical science part of the centre and also the larger Baltimore‐based research community. The latter is an interesting balancing act, which we’re trying to achieve in various innovative ways and with collaborative and interaction‐promoting activities. The distance is a hindrance, but COVID actually helped us in some ways: before that, it was difficult to join meetings and seminars in Baltimore, but that was solved instantaneously with the introduction of Zoom; we were actually more connected to Baltimore during lockdown than we have ever been, which has been a silver lining for us. We could not travel as much, but we’ve participated in many, many meetings.

## What was the most valuable advice you received during the early stages of your career?

The one piece of advice that was probably the most useful for me was when I was a postdoc in Peter Davies’ laboratory at the University of Texas in Houston. When I was finishing my postdoc, my plan was to go back to Hungary, start my laboratory, and perhaps collaborate with his laboratory, by visiting and sending students, and having a joint research programme. This was in the early 1990s, so things were not as connected as they are now – there was no e‐mail for instance, so my days were much more relaxed and I spent more time in the library than on Zoom! (*laughs*) And what Peter told me, which was very, very unselfish of him, was: ‘Laszlo, if you choose this path, it will be great for me; I would benefit from your knowledge, we would work together, and I’d get more papers out of this – but you wouldn’t learn much more. I’ve taught you everything I have to offer, but if you really want to complete your education, you need to go to a top‐tier, high‐pressure, high‐intensity laboratory to complete that’. And so he advised me to try to get into a superstar’s laboratory, which is what happened: I applied and got accepted into the laboratory of Ronald Evans, who is one of the discovers of the nuclear receptor superfamily. He had one of the prototypical large US laboratories with over thirty people and lots of interesting projects and research tools, some pressure and competition, but overall, it was a magical environment, sort of a time accelerator. Everything happened very fast. Joining his laboratory was an incredible experience for many reasons: obviously, I was lucky and I got good publications fast [5, 6, 8, 9], but what was really more important than that was I became part of an ecosystem, a large family of high‐profile researchers, including Ron as a mentor in the centre of this web and my peers, students, fellows and technicians. Later on, I realised that having the acquaintance and friendship of the other postdocs for the next 10–15 years of my career was in some ways the most important outcome of my training. I have forged life‐long friendships and scientific partnerships with some fellow post‐docs, such as John Schwabe (University of Leicester), Peter Tontonoz (UCLA) and Enrique Saez (Scripps), to name just the closest ones. So, the advice was ‘complete your training by going to the best lab possible,’ and now I can add to that, ‘where you can complete your training journey and get a great mentor, exciting projects, trusted friends and partners, and a scientific family which takes care of you for a long time’.

The other benefit of joining a high‐profile laboratory is it demystified the scientific process for me. When you are in a laboratory that’s not the absolute top notch and you open *Cell* or *Nature*, you see another paper from X laboratory, and you think there must be some high‐level geniuses there. When you actually join the laboratory, you realise the people there are like you: smart people, but not necessarily orders of magnitude smarter than you. This demystifies the process, and your self‐confidence grows almost regardless of your production there, which was also very important for me.

## What are your views on the future of scientific publishing, and what factors do you consider crucial for maintaining a successful scientific journal?

Well, this would be a long conversation – I can give you five main points:

First, I consider that scientific publishing is a business model which shouldn’t work, and yet it somehow works. It’s a broken model because the practising scientist does a disproportionate amount of the work: they get the funding, do the research, submit to the journal, pay for publication in the case of open‐access journals, review other people’s manuscripts for free, and pay to subscribe to subscription journals. If you presented this model to a bunch of capitalists, they would say that it shouldn’t work. This system is built on the vanity of scientists, who are driven to publish their paper in a journal with the highest possible impact factor and they do it without considering the economic cost. This situation needs to change, because for‐profit journals are taking advantage of and profiting from the work of researchers.

Second, for‐profit publishers are basically suffocating and overpowering society journals, including FEBS Press. Society journals are established by scientific organisations and run by scientists; yes, they employ professional editorial staff, but by and large, the journals are managed by practising scientists. That’s not true for most of the Nature Portfolio or Cell Press and some others. The problem is, with their financial might and practices, for‐profit journals are able to attract papers that would normally be submitted to society journals; my personal opinion is that journals such as *Cell Reports* and *Scientific Communications* represent the greatest competition, or if you like you can call it threat, to society journals because they receive thousands of submissions that would otherwise have gone to society journals. I think this is problematic because the profit goes into someone else’s pocket, rather than back to scientists, which is the case with FEBS Press, EMBO Press and many other society journals.

The third issue is, which I don’t have as strong an opinion on because I don’t know how to solve it, is the open‐access issue: I’m all for open access, and I understand that publishing costs money, but I think the inequities in the abilities of different authors to pay need to be balanced somehow.

My fourth point is that there are too many journals: my cynical opinion is that anything can be published, even if it’s in predatory journals that generate income by publishing anything that’s submitted there. A philosophical view, which is not mine but I endorse it, is that there should be just one journal: either something is publishable, or it is not. If anything can be published, then what is the value of publishing? Yes, we make a distinction between whether something can be published in *Nature* or in *FEBS Open Bio*, but I would like to believe that anything published in *FEBS Open Bio* is as valid as something published in *Nature*; maybe the former isn’t as exciting, or maybe it’s not so much of a breakthrough (at the moment, as things can change), but the basic criteria should be: based on what we know today, is this finding likely to be true or not? And if we have a situation wherein the upper 60% of journals are rigorous, but the bottom 30% are not, then why have the latter?

The fifth point is that we’re still publishing the same way our predecessors published a hundred years ago; we need to ask, what is a paper and how much data should it contain? Nowadays, a paper may include 5 figures and 55 supplementary figures. In my opinion, the primary data should be deposited so that other people can re‐analyse it. Publication should just be an advertisement: here are the data I acquired, and this is how I interpret it.

I can see some very positive changes in publishing, such as transparent peer review and publishing comprehensive datasets. I think with these innovations, we may be able to counter‐balance this spiral, in which more and more content is going to for‐profit journals. By opening up the constraints of a paper and making peer review transparent (I would still insist on having it anonymous, as people don’t act the same way if they have to sign their name to the review), we might be able to break down the tyranny of the for‐profit journals.

## What are the major differences between the United States and Europe in terms of securing public and private funding?

I think the major difference is that European systems favour past performance more than the actual proposal, while in the United States, it’s much more project‐based: I’ve been on NIH Study Sections, and I’ve realised, that if past performance reaches a certain threshold, that is, if the panel judges that you are someone who can do the research, it doesn’t really matter whether you have three *JCB* papers, or three *Nature* papers, you have the same fair shot at a grant. However, they are very rigorous when it comes to scrutinising what you’re proposing, and that’s because of the competition. So the style in which you write grants is very different; in Europe, at least in my experience, it’s more about expressing how good you are and how much you contribute to that particular field, while for the American system, you have to focus on the proposed research, rigour and reproducibility. So who benefits under these systems? Obviously more established investigators benefit in Europe than younger ones. While I like to believe that the US system is more permissive for younger people, I’m sure established researchers still also have the upper hand in the United States. Also, many countries in Europe and the EU itself also have mechanisms, such as the ERC, for which not only individual excellence, but also project excellence matters. National funding schemes tend to be more inbred and favour senior scientists.

The United States also has many more philanthropy‐based grants; there are entire systems, and it’s not just the big ones like Howard Hughes, there are also small foundation grants, various charities etc. The UK might be an exception here, but generally individual giving is a bigger thing in the United States than in Europe. There are many endowed chairs and individual donations, so people can build a grant portfolio that isn’t based entirely on state and federal funding, but also philanthropy, and that is largely missing in Europe. This is also true in many areas, not just science.

## What are your views on the future development of regional education, research and technology infrastructures in Hungary, and in East‐Central and South‐East Europe?

This is something I’ve been working on for many years and I am very passionate about, but the status quo is changing very slowly. It’s a grand idea everywhere, the United States included: you need to include more areas and have local centres. I’m currently on the EMBO Council so I can see that this divide is not diminishing at a sufficiently fast pace: a lot of money is poured into these regional centres and a lot of money is wasted. You need to have an infrastructure and you need to have good people there to absorb the funds. But the reality is that most people still want to go to Cambridge, or maybe London or Heidelberg, and not to Debrecen, not even Prague or Warsaw. So that’s changing slowly, but there are a few people who make this move. If we’re discussing central and eastern Europe, there is a lot of money available – some of my colleagues in Poland have said they’ve never been this rich in terms of money for research and infrastructure, so they can do better science than before. However, they have a hard time breaking down institutional boundaries: the evaluation system is not up to western standards, and they are certainly not internationalised sufficiently, so even if everything else is there – the money, the right people, the right structure to evaluate and promote them – there are very few foreigners to join those institutions. So I think more involvement from western institutions would help, by evaluating and promoting scientists, and sharing best practices. Locally, people who work in these countries need to convince their politicians that they need a different system, not one which is based on some obscure, ‘who knows who’ and ‘who likes who’ basis, but on objective evaluation and decision making with the help of outside experts. It’s hard, very, very hard – these countries, and Hungary is not an exception unfortunately, are moving backwards in some areas, not just in terms of science, but also in parts of the political system as well. At least relative to what I consider progress. I think we need to keep trying, but it’s going to take longer than I anticipated.

## Where would you rather spend your holidays: at lake Balaton or at Tampa Bay?

The answer is neither! Hungary and the entire state of Florida are very flat, so if I have an opportunity to go on vacation, I go to the mountains! I tend to alternate between Austria or Switzerland in Europe, or Colorado or North Carolina in the United States. Recent family holidays have tended to be in the Blue Ridge mountains in North Carolina or in Colorado because of my move and the pandemic, but if given the chance, I’d go to Austria. My holidays consist of hiking in the mountains, without computer access. I practically live on the beach in St. Petersburg, FL, so beach holidays aren’t particularly appealing to me.
